# Projection-specific Activity of Layer 2/3 Neurons Imaged in Mouse Primary Somatosensory Barrel Cortex During a Whisker Detection Task

**DOI:** 10.1093/function/zqaa008

**Published:** 2020-07-02

**Authors:** Angeliki Vavladeli, Tanya Daigle, Hongkui Zeng, Sylvain Crochet, Carl C H Petersen

**Affiliations:** 1 Laboratory of Sensory Processing, Brain Mind Institute, Faculty of Life Sciences, Ecole Polytechnique Fédérale de Lausanne (EPFL), Lausanne, Switzerland; 2 Allen Institute for Brain Science, Seattle, Washington, DC, USA

**Keywords:** neocortex, somatosensory cortex, projection neurons, sensory perception, whisker sensation, licking, goal-directed behavior, sensorimotor transformation, two-photon calcium imaging

## Abstract

The brain processes sensory information in a context- and learning-dependent manner for adaptive behavior. Through reward-based learning, relevant sensory stimuli can become linked to execution of specific actions associated with positive outcomes. The neuronal circuits involved in such goal-directed sensory-to-motor transformations remain to be precisely determined. Studying simple learned sensorimotor transformations in head-restrained mice offers the opportunity for detailed measurements of cellular activity during task performance. Here, we trained mice to lick a reward spout in response to a whisker deflection and an auditory tone. Through two-photon calcium imaging of retrogradely labeled neurons, we found that neurons located in primary whisker somatosensory barrel cortex projecting to secondary whisker somatosensory cortex had larger calcium signals than neighboring neurons projecting to primary whisker motor cortex in response to whisker deflection and auditory stimulation, as well as before spontaneous licking. Longitudinal imaging of the same neurons revealed that these projection-specific responses were relatively stable across 3 days. In addition, the activity of neurons projecting to secondary whisker somatosensory cortex was more highly correlated than for neurons projecting to primary whisker motor cortex. The large and correlated activity of neurons projecting to secondary whisker somatosensory cortex might enhance the pathway-specific signaling of important sensory information contributing to task execution. Our data support the hypothesis that communication between primary and secondary somatosensory cortex might be an early critical step in whisker sensory perception. More generally, our data suggest the importance of investigating projection-specific neuronal activity in distinct populations of intermingled excitatory neocortical neurons during task performance.

## Introduction

Neocortical neuronal activity is thought to contribute to sensory perception and volitional motor control, but the precise neuronal circuits underlying goal-directed sensory-to-motor transformations remain to be fully delineated. Investigation of head-restrained mice carrying out perceptual decision-making tasks, allows measurement of cell-type-specific activity in precisely defined brain regions. Mice gather important sensory information using their mystacial whiskers,^^1–4^^ and various whisker-dependent behaviors have been studied in head-restrained mice, including object localization^^5–9^^ and texture discrimination.^^10–12^^ Here, we investigate one of the simplest whisker-dependent tasks, namely stimulus detection, with perceived stimuli being reported through goal-directed licking.^^13–22^^ Previous work has found that neuronal activity in primary whisker somatosensory barrel cortex (wS1) correlates and causally contributes to task execution.^^15–18^^^,^^22^ Activity in wS1 might contribute to task execution through signaling to downstream targets.^4^^,^^23^ Excitatory neurons in layer 2/3 of wS1 project to two main cortical targets, the whisker primary motor cortex (wM1) and the secondary whisker somatosensory cortex (wS2).^10–12^^,^^^24–27^^ Previous work using whole-cell recordings targeted to retrogradely labeled neurons in layer 2/3 of wS1^21^ reported that wS2-projecting (S2p) neurons showed enhanced whisker-stimulus evoked depolarization compared to wM1-projecting (M1p) neurons, and that this developed across task learning. In addition, S2p neurons (but not M1p neurons) were found to depolarize before spontaneous licking after training.^21^ These data suggest that signals from wS1 to wS2 might be important for whisker detection. However, obtaining large datasets through whole-cell recordings is technically challenging,^28^ and typically both the numbers of recorded neurons and the numbers of trials per neuron are limited. In addition, *in vivo* whole-cell recordings are typically obtained one at a time (often in different animals), precluding simultaneous measurement of M1p and S2p neurons during the identical behavior. Two-photon imaging of neurons expressing genetically encoded calcium indicators, such as GCaMP6,^29^ allows measurement of neuronal network activity with cellular resolution over long periods of time during whisker-dependent head-restrained mouse behavior^7^^,^^8^^,^^10^ and can readily be combined with retrograde labeling of projection neurons.^10–13^ Previous such imaging studies during a whisker detection task found evidence supporting enhanced reciprocal signaling between wS1 and wS2,^13^^,^^22^ consistent with the electrophysiological study.^21^ However, these previous imaging studies during whisker detection tasks did not directly compare S2p and M1p neurons. Here, through dual-retrograde labeling and two-photon imaging of transgenic mice expressing GCaMP6f,^30^ we directly compare neuronal activity in S2p and M1p neurons during a whisker and auditory detection task, finding results consistent with our previous electrophysiological study.^21^ We advance current understanding through longitudinal imaging across days, finding relatively stable representations, and carrying out cross-correlation analyses, finding highly correlated spontaneous activity among S2p neurons.

## Materials and Methods

**Table:** 

Key Resources Table				
Reagent type (species) or resource	Designation	Source or reference	Identifiers	Additional information
Genetic reagent (*Mus musculus*)	*Short: Rasgrf2-dCre*	Jackson Laboratory	JAX: 022864	https://www.jax.org/strain/022864
	B6;129S-*Rasgrf2^tm1(cre/folA)Hze^*/J			
Genetic reagent (*Mus musculus*)	*Short: Tigre2-GCaMP6f (Ai148)*	Jackson Laboratory	JAX: 030328	https://www.jax.org/strain/030328
	B6.Cg-*Igs7^tm148.1(tetO-GCaMP6f,CAG-tTA2)Hze^*/J			
Software, algorithm	Matlab analysis code and data	*This paper*	Zenodo doi: 10.5281/	See Methods.
			zenodo. 3911112	Available from: https://doi.org/10.5281/zenodo.3911112

### Authorization for Animal Experiments

All experiments were performed in accordance with the Swiss Federal Veterinary Office, under authorization VD-1628 issued by the “Service de la consommation et des affaires vétérinaires” of the Canton de Vaud, Switzerland.

### Animal Preparation and Surgery

All experiments were carried out with 6- to 10-week-old female and male Rasgrf2-dCre mice^31^^,^^32^ crossed with TIGRE2.0 Cre-dependent GCaMP6f reporter mice (Ai148 mice).^30^ Recombinase activity of dCre was induced with trimethoprim antibiotic in 10% DMSO delivered by intraperitoneal injection (0.25 mg/g body weight) for 3 consecutive days.

Mice were deeply anesthetized with isofluorane gas anesthesia (3%–4% for induction) and then placed on the stereotaxic apparatus using a nose clamp. During surgery, the level of isoflurane concentration was maintained at 1.5%, temperature was controlled and held at 37°C with a heating pad (FHC Inc), and eyes were protected with an eye gel (VITA-POS, Pharma Medica AG). To prevent pain or inflammation after the surgery, mice were injected with carprofen intraperitoneally (0.3 mL at 0.5 mg/mL) (Rimadyl, Pfizer), and a mix of lidocaine (2% diluted 1:10) and bupivacaine (0.5% diluted 1:2) subcutaneously on the incision site before any surgical intervention. Furthermore, in order to avoid postoperative pain, ibuprofen was given in the drinking water for 3 days after surgery (2.5 mL in 250 mL of water bottle) (Algifor Dolo Junior, VERFORA SA). A povidone-iodine solution (Betadine, Mundipharma Medical Company) was used for skin disinfection before surgery. A part of the scalp was cut with surgical scissors and the skull was exposed. The membrane of the periosteum covering the skull was gently removed using a scalpel blade. The exposed skull was disinfected with Betadine, rinsed with Ringer solution, and then dried with cotton buds. Immediately after the bone was dried, a thin layer of cyanoacrylate glue was applied on the surface of the skull (Loctite 401, Henkel) and a small metal post was fixed onto the right hemisphere. Dental cement (Paladur, Kulzer) was added to reinforce the attachment of the head-post and create a chamber around the region of interest. A silicone elastomer (Kwik-Cast, WPI) was applied in the chamber to protect the exposed skull.

In order to target recordings to the C2 barrel column in wS1, intrinsic signal optical imaging was performed on the left hemisphere of mice immediately after head-post implantation. The level of anesthesia was maintained at 1% and temperature at 37°C. All whiskers except C2 were trimmed, and the animal was transferred on a holder to fix the head using the implanted metal head-post. The mouse head was placed under a CMOS camera coupled to a stereomicroscope (Leica MZ9.5) with a magnification of 3.2x. A reference image of the surface vasculature was first acquired under green illumination (525 nm, Thorlabs LED). Subsequently, the brain surface was illuminated with red light (630 nm, Thorlabs LED) continuously, and the right C2 whisker was inserted in a glass capillary attached to a piezoelectric actuator (PICMA, PI Ceramic). The C2 whisker was then deflected for 4 s in the antero-posterior direction at a repetition frequency of 10 Hz. Reflected light was collected through the stereomicroscope and recorded by the CMOS camera. This process was repeated several times, while trials without whisker stimulation were interleaved. The increase in absorption of red light upon tactile stimulation indicated the functional location of the C2 whisker in wS1 and wS2. Finally, the functional image of the intrinsic signal was overlaid on the anatomical image of the surface vasculature to map the C2 whisker representation relative to the surface blood vessels.

A triple glass window assembly was prepared before the surgery, which consisted of a 5 mm diameter coverslip attached to two 3 mm diameter coverslips of #1 thickness (CS-3R, Warner Instruments) using a UV-light curing adhesive (NOA61, Thorlabs), giving a total thickness of ∼0.45 mm. A small circular craniotomy of ∼3.5 mm in diameter was performed, while dura remained intact. The craniotomy was centered on the C2 barrel column in wS1 and included wS2. Cholera toxin subunit-B (CTB) conjugated with Alexa-Fluor 594 was then injected into wS2 (Molecular Probes, Invitrogen; 100 nL, 0.5%, wt/vol) using a glass pipette (tip diameter = 27–30 µm) . Injection volume was 50 nL at 300 μm and 50 nL at 500 μm below the pial surface, giving a total injection volume of 100 nL. After CTB injection, the triple coverslip was then placed in the craniotomy, and permanently sealed and fixed to the skull using UV-curing adhesive (Thorlabs, NOA68). A second craniotomy of ∼1 mm in diameter, was then opened over the wM1 using stereotaxic coordinates (1 mm anterior, 1 mm lateral to Bregma). CTB conjugated with Alexa-Fluor 647 (Molecular Probes, Invitrogen; 200 nL, 0.25%, wt/vol) was injected into wM1 following the same procedure (100 nL at 300 μm and 100 nL at 500 μm below the pial surface; total injected volume: 200 nL) . In some mice, CTB injections were inverted, such that CTB Alexa-Fluor 647 was injected into wS2 and CTB Alexa-Fluor 594 was injected into wM1. A small amount of Kwik-Cast was then applied to protect the craniotomy. Finally, UV-curing adhesive (Thorlabs, NOA68) and dental cement were applied on top to seal the craniotomy and provide stability to the cranial window over the long term. Mice were allowed to recover for a minimum of 1 week after the surgery.

**Figure 1.**
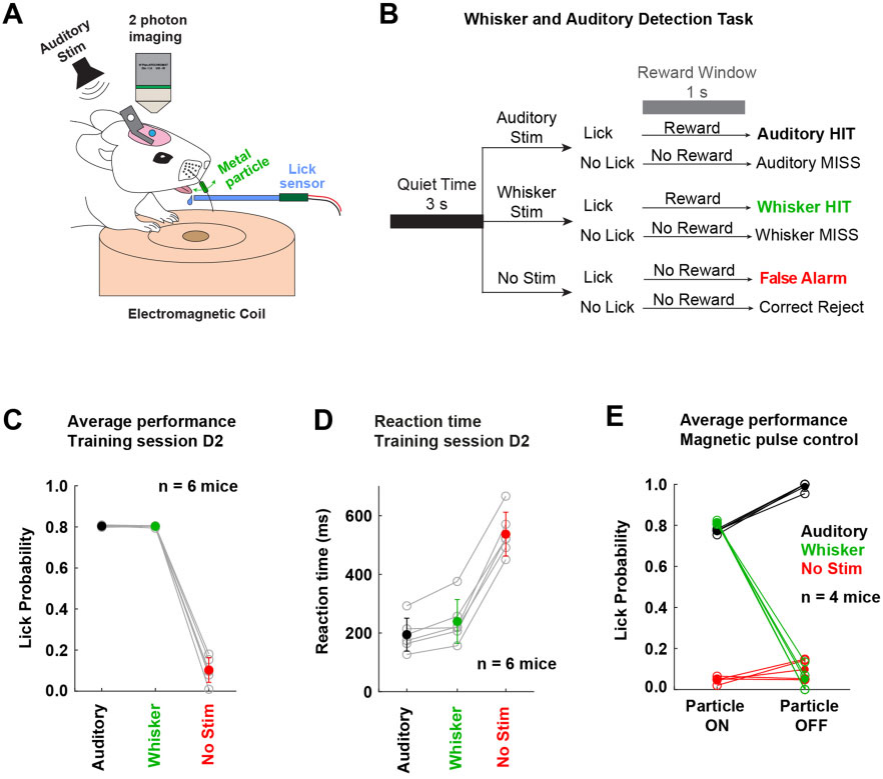
A Whisker and Auditory Detection Task for Head-restrained Mice. (**A**) Schematic of experimental setup. (**B**) Trial types included auditory hit (AHIT), auditory miss (AMISS), whisker hit (WHIT), whisker miss (WMISS), false alarm (FA), correct rejection (CR) trials. (**C**) Performance of six expert mice. (**D**) Quantification of the reaction time from stimulus onset (time from stimulus presentation until the first lick). (**E**) Expert mice were exposed to a control experiment removing the metal particle from the whisker to test whether the magnetic pulse acted specifically via the metal particle attached to the C2 whisker, and not via other potential cues. Performance on whisker trials was high when the metal particle was on the whisker (Particle ON), and almost abolished when the metal particle was removed from the whisker (Particle OFF).

### Behavioral Training

Head-fixed mice were trained in a go/no-go whisker and auditory detection task.^16^ Approximately 8–10 days after surgery, mice were subjected to water restriction. Sensory percepts were reported by licking of a water spout attached to a piezo sensor that activates the delivery of a water drop (4–5 μL) through an electromagnetic valve. Mice were initially habituated to head-restraint on the recording set-up and exposed to “free-licking” for 1–2 sessions (pre-training phase). In “free-licking” sessions, mice received water drops at random time points, in order to engage in licking from the spout. Subsequently, mice were trained to lick the spout for water reward in response to detected auditory and whisker stimuli presented in randomly interleaved trials. To measure the spontaneous licking rate, trials in which no stimulus was delivered (no-stim catch trials) were interleaved with other trials. When mice had reached high and stable performance in the detection of both sensory modalities, they were subjected to a whisker particle control experiment (post-training phase). In the control experiment, training on the whisker and auditory detection task started as usual, and after ∼100 trials the iron particle attached to the whisker was removed; therefore, there was no whisker sensory input to the animal. Mice performed under this condition for a block of ∼100 trials, and then the iron particle was attached again to the whisker, and mice continued performing until they stopped licking for a reward.

**Figure 2.**
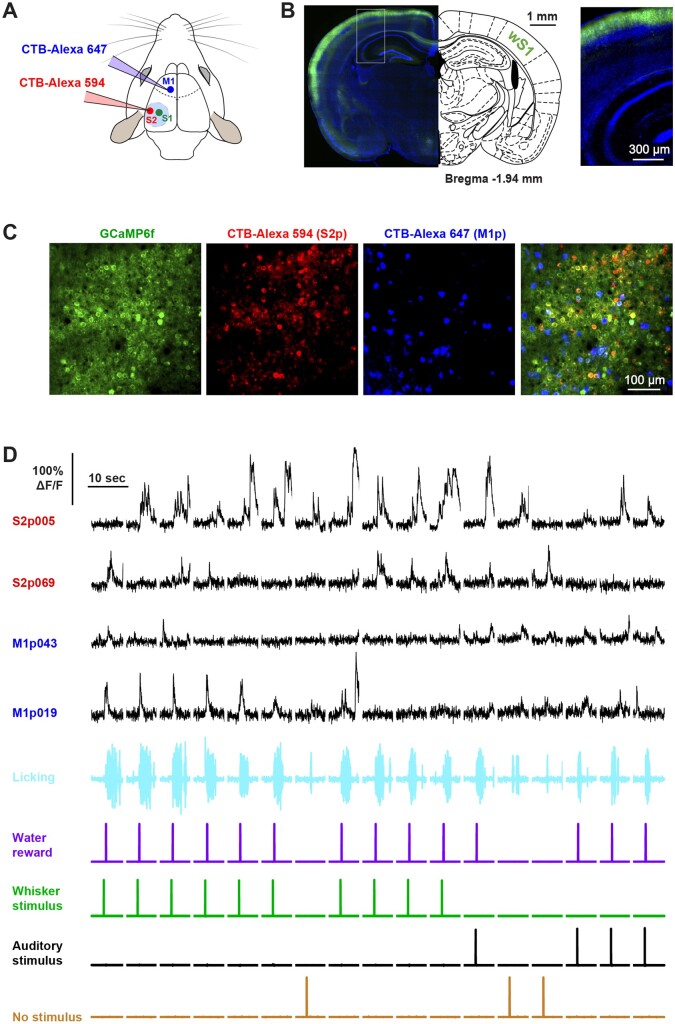
Two-Photon Calcium Imaging of Layer 2/3 Projection Neurons. (**A**) Retrograde labelling of S2p and M1p neurons in wS1. Schematic of mouse brain showing retrograde tracers and injection areas. CTB conjugated with AlexaFluor-594 (red) and AlexaFluor-647 (blue) was injected into wS2 and wM1 of the left hemisphere. (**B**) L2/3 neurons selectively expressed GCaMP6f in Rasgrf2-dCre mice crossed with TIGRE2.0 Cre-dependent GCaMP6f reporter mice (Rasgrf2-dCre x Ai148). Example coronal brain section around the center of whisker primary somatosensory cortex (left panel) aligned according to a mouse brain atlas,^33^ with a higher magnification image of cortex (right panel). (**C**) CTB-labeled layer 2/3 neurons in wS1 at the subpial depth of 200 μm imaged in vivo with a two-photon microscope. S2p neurons (red) were labeled with CTB AlexaFluor-594, M1p neurons (blue) were labeled with CTB AlexaFluor-647, and in green neurons expressing GCaMP6f. (**D**) GCaMP6f fluorescence traces of two S2p and two M1p neurons imaged simultaneously during task performance.

For auditory stimulation, a brief auditory tone of 10 ms duration was added (10 kHz pure tone of 3 dB) on top of a continuous white noise sound (80 dB). The white noise sound was used throughout training to mask any possible background sounds. For whisker stimulation, a small iron particle was attached to the whisker and deflected by a rapid change of a magnetic field. A magnetic coil was placed under the mouse head centered on the right C2 whisker. The coil produced magnetic pulses of 1 ms duration that vertically deflected the whisker. Trials with either whisker or auditory stimulation, as well as catch trials were presented at random inter-stimulus intervals ranging from 11 s to 14 s. Catch trials were presented with 30%–40% probability of all trials. A trial consisted of a 3-s pre-stimulus period (quiet time) followed by stimulus delivery. If mice licked within the 3 s quiet time window, the trial was aborted and a 5–8 s time out period was imposed on the mouse. The time window during which mice could be rewarded was 1 s after stimulus delivery. Control and data acquisition of behavior experiments were performed using a custom-written LabVIEW software (National Instruments).

In the whisker and auditory detection task, there were six trial types. When licking occurred immediately after auditory stimulation (within 1 s reward window), water was delivered to the mouse and the trial characterized as “auditory hit.” When mice did not lick after auditory stimulation, the trial scored as “auditory miss.” Similarly, in whisker stimulation trials, mice were rewarded with water delivery if licking occurred after whisker deflection (within 1 s reward window), and it was defined as “whisker hit” trial, but if there was no licking, the trial was scored as “whisker miss.” During catch trials, if there was no licking, the trial was characterized as “correct rejection,” and if there was licking (within 1 s reward window from a virtual stimulus time), the trial was scored as “false alarm.”

### Two-Photon Imaging

A custom made two-photon laser scanning microscope was built to perform chronic calcium imaging. Images were acquired at a frequency of 30 Hz with 512 x 512 pixel resolution. A tunable near-infrared laser (MaiTai DeepSee, Spectra-Physics) was used for two-photon excitation (940 nm for GCaMP6; 800 nm for AlexaFluor-594 and AlexaFluor-647). A Pockels cell (model 302RM, Conoptics) was used to control the laser beam intensity. The scanning system consisted of an 8 kHz resonant scanner (CRS Series, Cambridge Technology, GSI) coupled to a galvo scanner (M-Series, model 6210, Cambridge Technology, GSI). A 20x water immersion objective (W Plan-Apochromat 20x/1.0 DIC, Zeiss) was used for functional imaging through the cranial window. A dichroic mirror (705 nm edge BrightLine (FF705-Di01-25x36), Semrock) was used to transmit the excitation light and reflect the emitted fluorescence photons, which then pass through an infrared blocker (blocking from 770 nm to 1100 nm) (ET750sp-2p, Chroma). Two GaAsP photodetectors (H10770PA-40, Hamamatsu) were used to collect the emitted fluorescence signals. Two different removable filter cubes (DFM1, Thorlabs) were used in order to achieve 3-color fluorescence detection (green, red, and far red): one coupled to emission filters for green and red wavelengths (green filter for GCaMP6: 510/84 nm BrightLine (FF01-510/84-25); red filter for AlexaFluor-594: 607/70 nm BrightLine (FF01-607/70-25); dichroic mirror: 562 nm edge BrightLine (FF562-Di03-25x36), Semroch), and one coupled to emission filters for red and far-red wavelengths (far-red filter for AlexaFluor 647: 700/75 nm Chroma (ET700/75m); red filter for AlexaFluor-594: 609/57 nm BrightLine (FF01-609/57-25); dichroic mirror: 649 nm edge BrightLine (FF649-Di01-25x36), Semrock). PMT signals were amplified using a variable gain high-speed current amplifier (DHPCA-100, Femto), digitized using an A/D converter (NI-5732, National Instruments) and transmitted to an FPGA module (NI PXI-7813R, National instruments), which was integrated into a chassis (NI PXIe-1073, National instruments). The two-photon microscope was controlled through Matlab-based software (ScanImage 5, Vidrio Technologies).^34^

Chronic imaging started at earliest 8 days after cranial window implantation. Brain surface vasculature was imaged through the cranial window using a wide-field camera that was aligned with the two-photon microscope, in order to identify the center of C2 barrel column according to the intrinsic signal optical imaging map. Imaging was switched to two-photon mode and a z-stack at the center of C2 barrel was acquired of about 250 μm from the surface with 5–10 μm steps. The imaging field-of-view (FOV) was chosen on the basis of the location of S2p and M1p neurons at the center of C2 barrel and was the same across all training sessions for each animal. An average image of the selected FOV was created before the start of the first training session, which was used as the master reference image to find the same imaging location at every session. During imaging session data were acquired continuously (gap-free). The behavior control program provided three analog input signals 0–5 V (Start, Stop, Next) to ScanImage in order to trigger the start of image acquisition, the next file opening to save trial data and the end of acquisition.

### Two-Photon Calcium Data Processing

Calcium imaging data analysis was performed using Matlab (MathWorks). Anatomical fluorescence data acquired from all three channels (GCaMP6f, AlexaFluor-594, AlexaFluor-647) was imported into Matlab for processing. Movies were corrected for movement artifacts (2-D rigid translation),^35^ and average images for each channel were created. A 3-channel stack image containing red fluorescent AlexaFluor-594 (S2p neurons in red channel), far-red AlexaFluor-647 (M1p neurons in blue channel), and GCaMP6f was formed, which was used as a master reference image.

Functional imaging data acquired from the green channel (GCaMP6f) was imported into MATLAB for processing. The first step of processing was the subtraction of background, which was defined as the minimum fluorescence value across the entire video for each trial. Motion correction was performed using a Matlab routine, which performs subpixel image registration by cross-correlation (2-D rigid translation).^35^ Regions of interest (ROIs) corresponding to individual neurons were manually selected from the master reference image of the first imaging session using ImageJ (ImageJ, National Institutes of Health, USA). For each session, a reference mean image over a number of frames (∼2000–3000 frames) was created. Reference images of each session were then registered (affine transformation consisting of translation, rotation, scale, and shear) to the master reference image of the first session, and the calculated transformations were stored. The selected ROIs were then registered on the reference image of each session by applying the calculated transformations. Intensity values of all pixels within each ROI were averaged and extracted for each frame across all imaging sessions. Neuropil contamination was corrected by subtracting the fluorescent signal from a surrounding ring from somatic fluorescence, and was measured in ∼40 μm radius around the center of each neuron, excluding any detected ROIs. The corrected fluorescence signal of a neuron was calculated as:

$$F_{\mathrm{corrected}}=F_{\mathrm{soma}}-\;a_{\mathrm{neuropil}}(F_{\mathrm{neuropil}}-\;{\mathrm{median}(F}_{\mathrm{neuropil}}))$$

where $a_{\mathrm{neuropil}}$ is the coefficient of neuropil contamination, which was set to 0.7.^36^ Calcium signals were expressed as relative change in fluorescence $\Delta F/F=\left(F-F_{o}\right)/F_{o}$. $F_{o}$ was calculated as the mean value of 60 frames before the trial start (the stimulus or no-stimulus event). The degree of correlation of activity in simultaneously imaged S2p and M1p neurons for correct rejection trials was computed by first subtracting the mean value of ΔF/F for each ROI across trials and then applying the MATLAB function xcorr between pairs of neurons.

## Statistics

Data were represented as mean ± SD unless otherwise noted. The Wilcoxon signed-rank test was used to assess significance in paired comparisons, and the Wilcoxon rank-sum test was used for unpaired comparisons. The statistical tests used and *n* numbers are reported explicitly in the main text or figure legends.

## Data and code availability

The data and Matlab analysis code for generating the figures are freely available at the CERN database Zenodo: https://doi.org/10.5281/zenodo.3911112.

## Results

### An Auditory and Whisker Detection Task

Six head-restrained mice were trained to lick a reward spout in response to randomly interleaved presentations of either a brief deflection of the C2 whisker or a brief auditory tone (Figure 1A). A drop of water was delivered if the mouse licked within 1 s of either of the two sensory stimuli (Figure 1B). Catch trials without stimuli were interleaved to assess spontaneous false alarm licking (Figure 1B). After several days of training, mice reached stable performance, responding equally to whisker or auditory stimuli with low false alarm rates (Figure 1C). Lick reaction time was short in trials with sensory stimulation (195 ± 56 ms for auditory hit trials; 240 ± 74 ms for whisker hit trials; mean ± SD; *n* = 6 mice), and longer in catch trials (537 ± 75 ms; *n* = 6 mice) (Figure 1D). Whisker deflection was driven by 1-ms duration magnetic pulses acting on a metal particle attached to the C2 whisker.^14–16^^,^^18^^,^^19^^,^^21^ In order to test if the magnetic pulse evoked licking through whisker deflection, rather than any other potential cues, we removed the metal particle from the whisker as a control. In these “particle off” sessions, licking in response to the magnetic pulse dropped to the false alarm rate, whereas licking in response to the auditory stimulus remained at a high level (particle on: magnetic pulse whisker hit rate 81.2 ± 0.8%, auditory hit rate 77.1 ± 1.0%, false alarm rate 4.6 ± 1.6%; particle off: magnetic pulse whisker hit rate 5.1 ± 5.6%, auditory hit rate 98.9 ± 2.0%, false alarm rate 9.8 ± 4.8%; *n* = 4 mice) (Figure 1E). Mice therefore did not detect the magnetic pulse directly, but rather detected the induced deflection of the C2 whisker, performing equally well for whisker and auditory detection.

### Two-Photon Calcium Imaging of Retrogradely Labeled Neurons

Before training the mice, projection neurons in wS1 were retrogradely labeled by injecting CTB conjugated to different Alexa fluorophores in wM1 and wS2. In some experiments, CTB AlexaFluor-594 was injected into wS2 and CTB AlexaFluor-647 was injected into wM1 (Figure 2A) and in other experiments, the colors were reversed. We did not observe differences in results depending upon which Alexa dye was injected in which region, and the results were therefore pooled. We used transgenic mice expressing GCaMP6f in layer 2/3 neurons through crossing Ai148 TIGRE2.0-GCaMP6f mice^30^ with Rasgrf2-dCre mice^31^ (Figure 2B). We imaged neuronal activity at 30 Hz in wS1 through a cranial window^37^ using a resonance scanning two-photon microscope. Using different bandpass emission filters and different excitation wavelengths, we could separately image GCaMP6f, AlexaFluor-594, and AlexaFluor-647 signals, allowing unequivocal identification and functional imaging of neurons projecting to wS2 and wM1 (Figure 2C). Some projection neurons appeared to show task-related calcium signals (Figure 2D). Across the six mice, in total, we imaged 172 S2p neurons (29 ± 8 S2p neurons per mouse, range 19–39 S2p neurons) and 154 M1p neurons (26 ± 10 M1p neurons per mouse, range 19–44 M1p neurons).

### Neurons Projecting to wS2 Have Larger Sensory-evoked Responses Compared to Neurons Projecting to wM1

**Figure 3.**
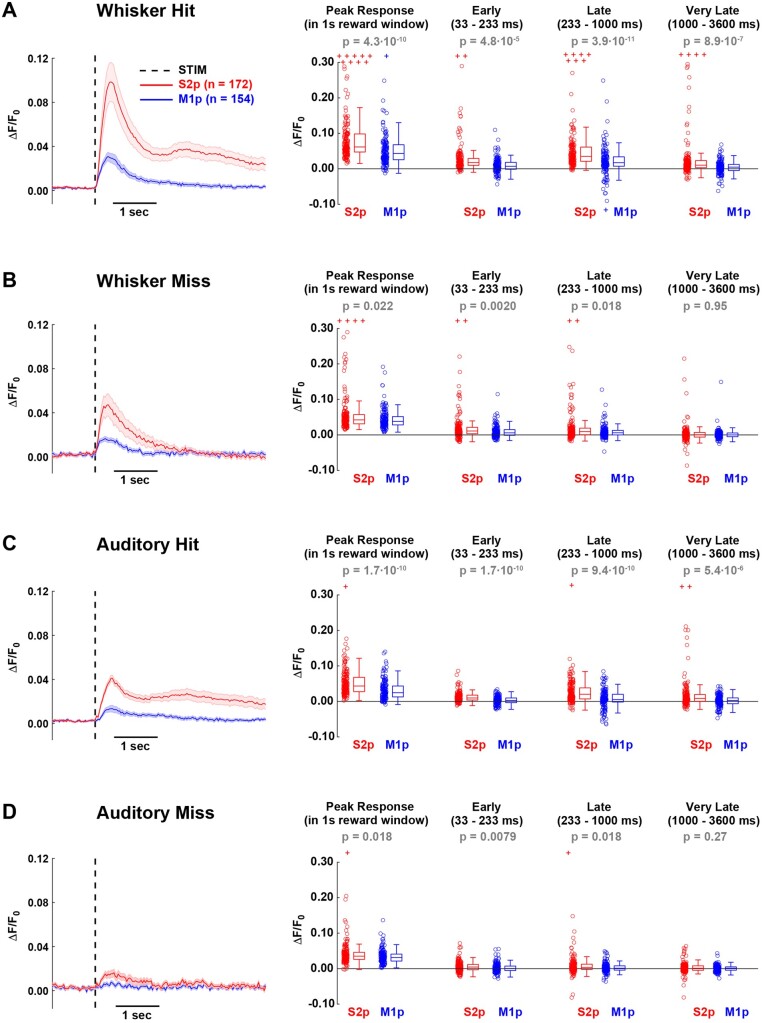
GCaMP6f Signals in S2p and M1p Neurons in L2/3 of wS1 During Task Performance for (**A**) whisker hit, (**B**) whisker miss, (**C**) auditory hit and (**D**) auditory miss trials. Left panel shows the grand average response across all S2p and M1p neurons (thick line: mean, shading: ± SEM). Quantification of mean peak response within the 1 s reward window, as well as mean response in different time windows after stimulus presentation: early (33–233 ms), late (233–1000 ms), and very late (1000–3600 ms). In the box plots, central line indicates the median, and bottom and top edges of the box indicate the 25th and 75th percentiles, respectively. The whiskers show the most extreme data points not including outliers, and off-scale outliers are indicated using the “+” symbol. Open circles represent individual neurons. Wilcoxon rank-sum test was used for statistical comparisons.

We aligned the GCaMP6f fluorescence traces to the onset of sensory stimuli and averaged within trial-types across a single behavioral session (same session as quantified in Figure 1C, D) of well-trained mice (Figure 3). In trials in which the mice licked in response to whisker stimulation (whisker hit trials), S2p neurons showed larger amplitude responses compared to the simultaneously imaged M1p neurons (Figure 3A). The early sensory response quantified from 33 ms to 233 ms post-stimulus was significantly larger in S2p neurons compared to M1p neurons (ΔF/F_0_ S2p: 0.036 ± 0.094, *n* = 172 cells; ΔF/F_0_ M1p: 0.014 ± 0.019, *n* = 154 cells; Wilcoxon rank-sum test *P* = 4.8 x 10^−5^). Later activity (quantified from 233 ms to 1000 ms) was also significantly larger in S2p neurons compared to M1p neurons (ΔF/F_0_ S2p: 0.070 ± 0.175, *n* = 172 cells; ΔF/F_0_ M1p: 0.019 ± 0.041, *n* = 154 cells; Wilcoxon rank-sum test *P* = 7.3 x 10^−9^). Some neurons had long-lasting calcium signals and this very late activity (quantified from 1000 ms to 3600 ms) was also significantly enhanced in S2p compared to M1p neurons (ΔF/F_0_ S2p: 0.031 ± 0.075, *n* = 172 cells; ΔF/F_0_ M1p: 0.004 ± 0.018, *n* = 154 cells; Wilcoxon rank-sum test *P* = 8.9 x 10^−7^). The peak response amplitude within the 1-s reward window was also larger for S2p neurons compared to M1p neurons (ΔF/F_0_ S2p: 0.117 ± 0.237, *n* = 172 cells; ΔF/F_0_ M1p: 0.052 ± 0.048, *n* = 154 cells; Wilcoxon rank-sum test *P* = 4.3 x 10^−10^). S2p neurons also responded with significantly larger calcium signals compared to M1p neurons in whisker stimulus trials in which the mouse failed to lick (whisker miss trials) (Figure 3B) for early, late, and peak response, but not for the very late period 1000–3600 ms after the whisker stimulus. Although the auditory stimulus evoked a smaller response, the overall pattern of activity comparing S2p and M1p neurons was similar to that in whisker stimulus trials. S2p neurons had larger evoked responses compared to M1p neurons in auditory hit trials across all quantified epochs (Figure 3C), and in auditory miss trials S2p neurons had larger evoked responses in early, late, and peak responses, but not in the very late period (Figure 3D).

**Figure 4.**
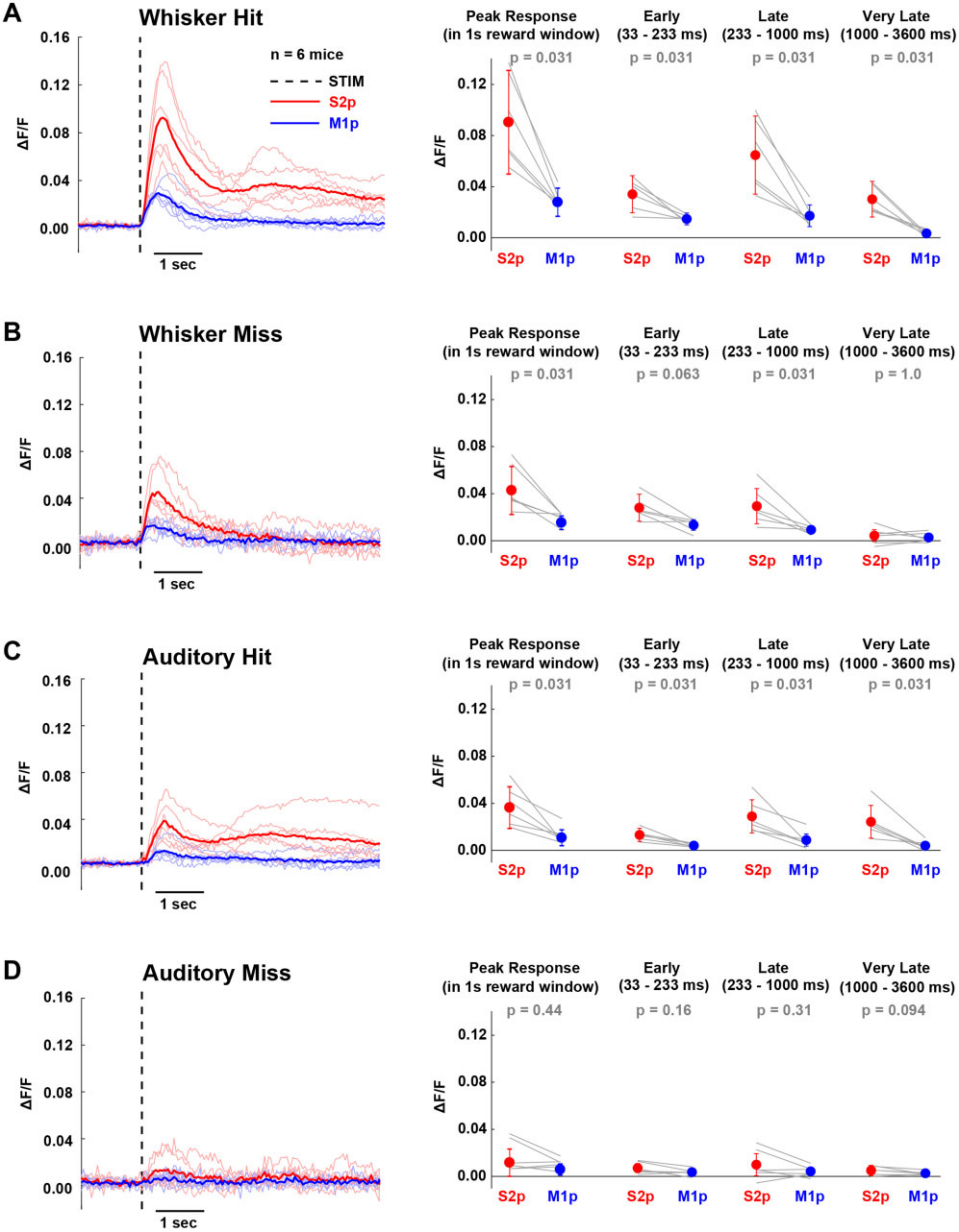
Distinct GCaMP6f Signals in S2p versus M1p Neurons Were Found in Each Individual Mouse for (**A**) whisker hit, (**B**) whisker miss, (**C**) auditory hit and (**D**) auditory miss trials. Thick lines correspond to the population means across mice and thin lines to average responses of individual animals. Dashed line indicates stimulation event. Quantification of mean peak response within the 1 s reward window, as well as mean response in different time windows after stimulus presentation: early (33–233 ms), late (233–1000 ms) and very late (1000–3600 ms). Wilcoxon signed-rank test was used for statistical comparisons.

The projection neurons were recorded across six mice, and in order to test for the robustness of our results across mice, we separately computed the average responses for S2p and M1p neurons in each mouse (Figure 4). We again found that S2p neurons had significantly larger calcium signals in whisker hit trials than M1p neurons in early, late, very late, and peak responses (Figure 4A). In whisker miss trials, we found that the late and peak responses were significantly larger in S2p compared to M1p neurons (Figure 4B). In auditory hit trials, all periods quantified showed significantly larger responses in S2p compared to M1p neurons (Figure 4C). No significant differences were found in auditory miss trials (Figure 4D).

**Figure 5.**
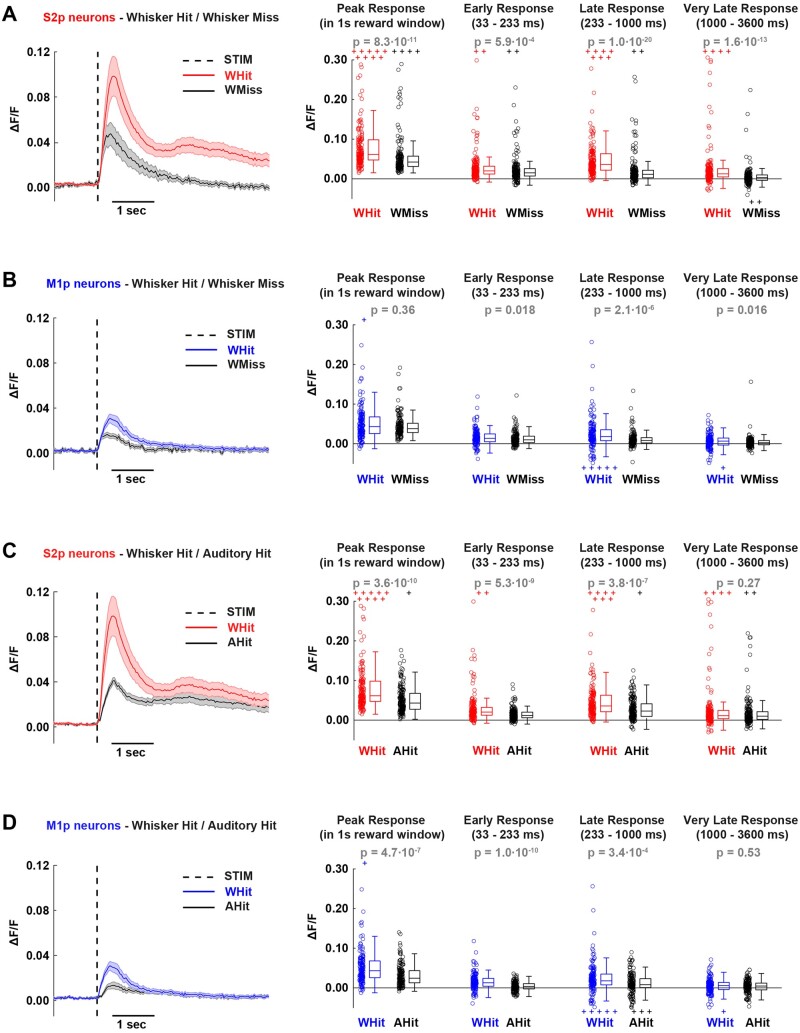
Neuronal Responses Correlated with Task Execution for S2p and M1p Neurons in L2/3 of wS1. (**A**) Left panel, grand average traces of S2p neurons (thick line: mean, shading: ± SEM) during hit (red) and miss (black) trials. Right, quantification of mean peak response within the 1 s reward window, as well as mean response in different time windows after stimulus presentation: early (33–233 ms), late (233–1000 ms), and very late (1000–3600 ms). In the box plots, central line indicates the median, and bottom and top edges of the box indicate the 25th and 75th percentiles, respectively. The whiskers show the most extreme data points not including outliers, and off-scale outliers are indicated using the “+” symbol. Open circles represent individual neurons. Wilcoxon rank-sum test was used for statistical comparisons. (**B**) Same as A, but for M1p neurons. (**C**) and (**D**) Same as A and B, respectively, but for whisker hit versus auditory hit trials.

We next compared across trial types within each class of projection neuron (Figure 5). We first compared trials where the same stimulus was delivered (whisker) but with different motor output (hit vs. miss). For S2p neurons, whisker hit trials had significantly larger calcium signals compared to whisker miss trials in early, late, very late, and peak responses (Figure 5A). M1p neurons also had significantly larger responses in whisker hit compared to whisker miss trials when quantified in early, late, and very late periods, but not for the peak response within the 1-s reward window (Figure 5B). We next compared trials in which the mouse performed the same motor output (hit trials) in response to different sensory stimuli (whisker vs. auditory). The early, late, and peak responses in both S2p (Figure 5C) and M1p (Figure 5D) neurons were larger for whisker hit compared to auditory hit trials. However, the very late calcium signals were not significantly different for either S2p or M1p neurons comparing whisker hit to auditory hit trials. The very late calcium activity might therefore mainly reflect licking-related signals.

### Neurons Projecting to wS2 are More Excited During Licking Compared to Neurons Projecting to wM1

**Figure 6.**
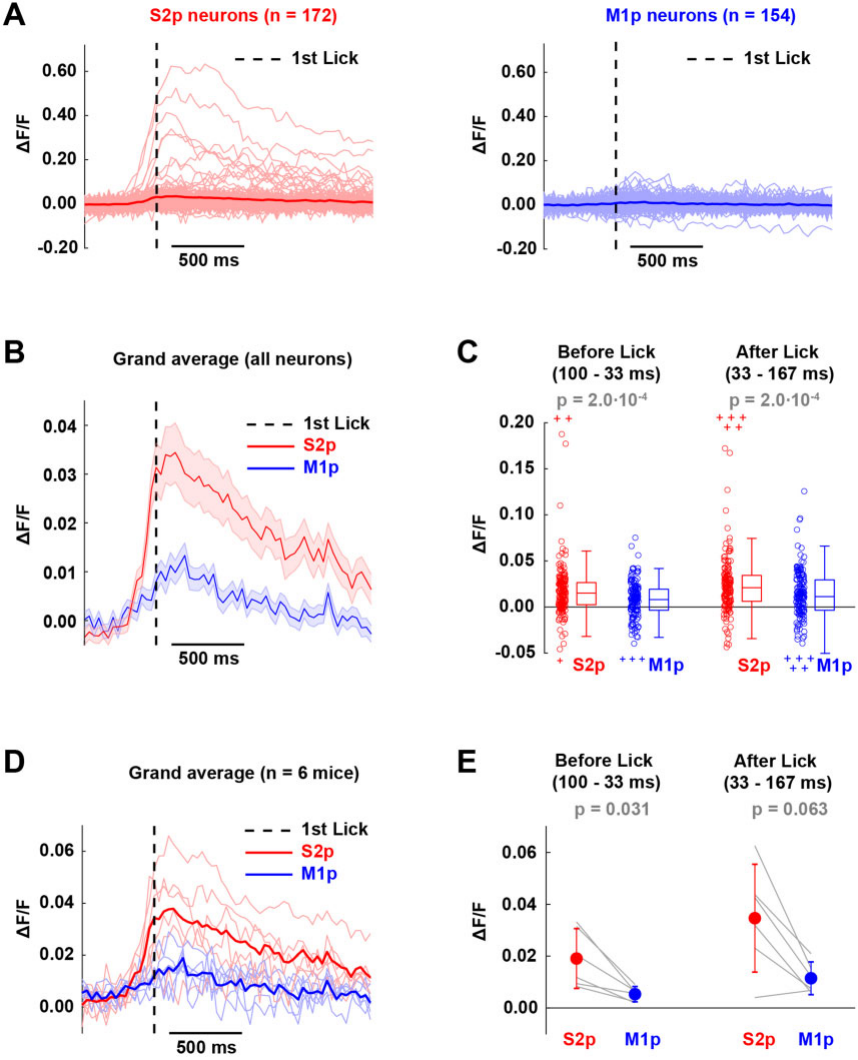
Lick-triggered Analysis in False Alarm Trials. (**A**) Average calcium responses aligned to lick onset for individual neurons (thin lines) and for population means (thick solid lines) of S2p and M1p neurons in L2/3 of wS1. Dashed line indicates 1st lick time. (**B**) Grand average response of S2p and M1p neurons (thick line: mean, shading: ± SEM). (**C**) Quantification of mean response before (100–33 ms) and after (33–167 ms) the 1st lick time for individual neurons. In the box plots, the central line indicates the median, and the bottom and top edges of the box indicate the 25th and 75th percentiles, respectively. The whiskers show the most extreme data points not including outliers, and off-scale outliers are indicated using the “+” symbol. Open circles represent individual neurons. Wilcoxon rank-sum test was used for statistical comparisons. (**D**) Grand average lick-triggered response computed across six mice for S2p and M1p neurons in L2/3 of wS1 for false alarm trials. Thick lines correspond to population mean across mice and thin lines to average responses of individual animals. (**E**) Quantification of mean response before (100–33 ms) and after (33–167 ms) the first lick time. Wilcoxon signed-rank test was used for statistical comparisons.

Having investigated the calcium signals in trials with sensory stimuli, we next examined false alarm trials (Figure 6). In these trials, no sensory stimulus was delivered, but the mouse spontaneously initiated licking, which was unrewarded. We aligned the GCaMP6f traces to the first lick time and averaged across all false alarm trials. Whereas some S2p neurons showed a prominent lick-triggered calcium signal, there appeared to be much less modulation of M1p neurons (Figure 6A). Averaged across neurons, S2p neurons showed a clear increase in calcium starting hundreds of milliseconds before lick time, and the signal in S2p neurons appeared considerably larger than M1p neurons (Figure 6B). Quantified across the 100-33 ms before lick-time and in the 33-167 ms after lick-time relative to a baseline window 1200-466 ms before lick-time, S2p neurons showed significantly larger increases in calcium compared to M1p neurons (Figure 6C). The same measures computed across mice showed similar dynamics (Figure 6D), with a significantly larger signal in S2p neurons compared to M1p neurons for the 100-33 ms before lick-time window (Figure 6E).

### Neuronal Responses are Relatively Stable Across Days

**Figure 7.**
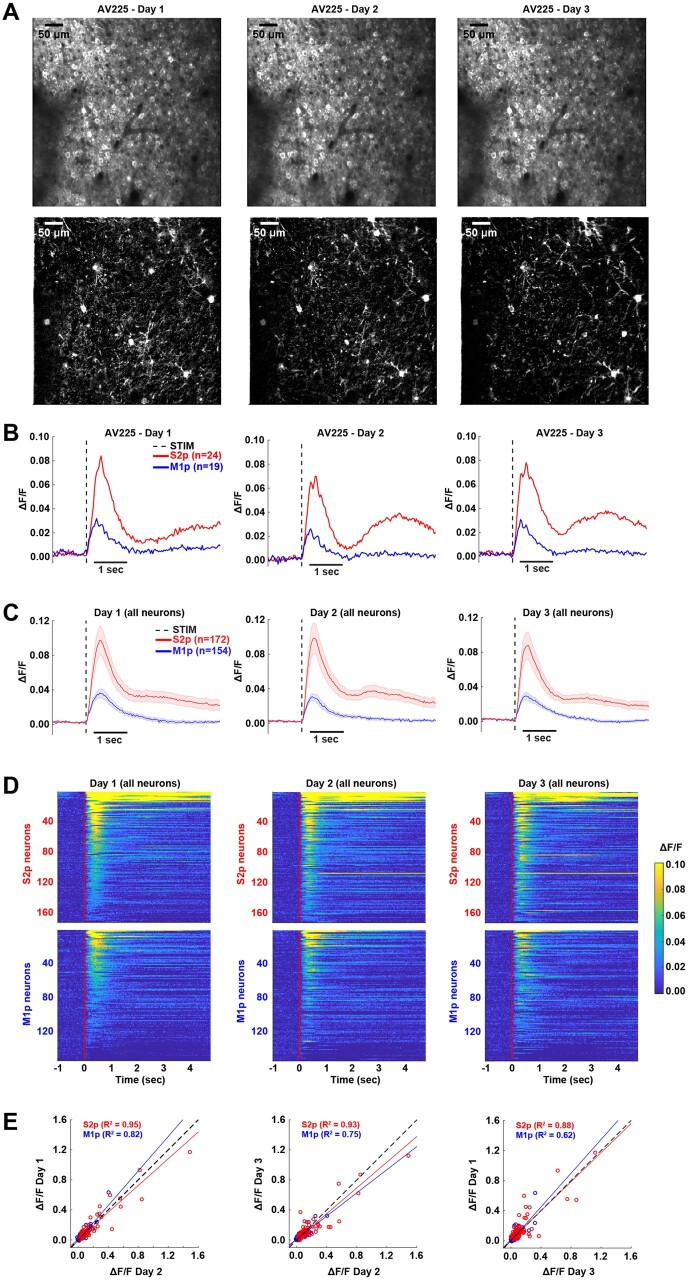
Longitudinal Monitoring of Calcium Signals in S2p and M1p Neurons During Task Performance. (**A**) Top panel: stable recordings over three training sessions (Day 1, Day 2, and Day 3) of an example mouse (AV225). Average two-photon image of the recorded field-of-view across sessions. Bottom panels: maximum projection ΔF/F image during 1 s after stimulation over days for whisker hit trials. (**B**) Average GCaMP6f responses of S2p and M1p neurons for whisker hit trials across sessions for the example mouse. (**C**) Grand average response of all S2p and M1p neurons across sessions (thick line: mean, shading: ± SEM). (**D**) Heat map of GCaMP6f signals for each neuron in whisker hit trials across sessions. Each line of the image corresponds to the average calcium trace of an individual neuron. (**E**) Comparison of calcium responses of S2p and M1p neurons between sessions. A least-squares linear fit was superimposed on each scatter plot, and the *R*-squared coefficient is indicated for each cell type. The dashed line at 45° refers to the regression line of unity.

We investigated the stability of neuronal activity by imaging the same field-of-view over 3 consecutive days for each mouse (Figure 7). In an example mouse (Figure 7A, B), as well as in the other mice, many neurons could be readily identified across all three imaging sessions. The overall spatial activity patterns in whisker hit trials appeared similar on each day (Figure 7A), along with similar average calcium dynamics across days, with the S2p neurons having larger and longer-lasting responses than the M1p neurons in the example mouse (Figure 7B). Similarly, analyzing all neurons across the six mice, we found the grand average population dynamics in whisker hit trials also appeared stable across the 3 days (Figure 7C). Ranking the neurons according to response amplitude in whisker hit trials on Day 2 with a color-coded heat-map, we found a similar pattern of activity across days, with obviously enhanced signals in S2p neurons compared to M1p neurons (Figure 7D). For quantitative comparison, we plotted the peak response amplitude in whisker hit trials for each cell comparing across days, finding that data points were typically close to the line of unity (Figure 7E). For S2p neurons, the explained variance (*R*^2^) was 0.95 for Day 1 versus Day 2, 0.93 for Day 3 versus Day 2, and 0.88 for Day 1 versus Day 3. For M1p neurons, the explained variance (*R*^2^) was 0.82 for Day 1 versus Day 2, 0.75 for Day 3 versus Day 2, and 0.62 for Day 1 versus Day 3.

### Highly Correlated Spontaneous Activity among S2p Neurons

**Figure 8.**
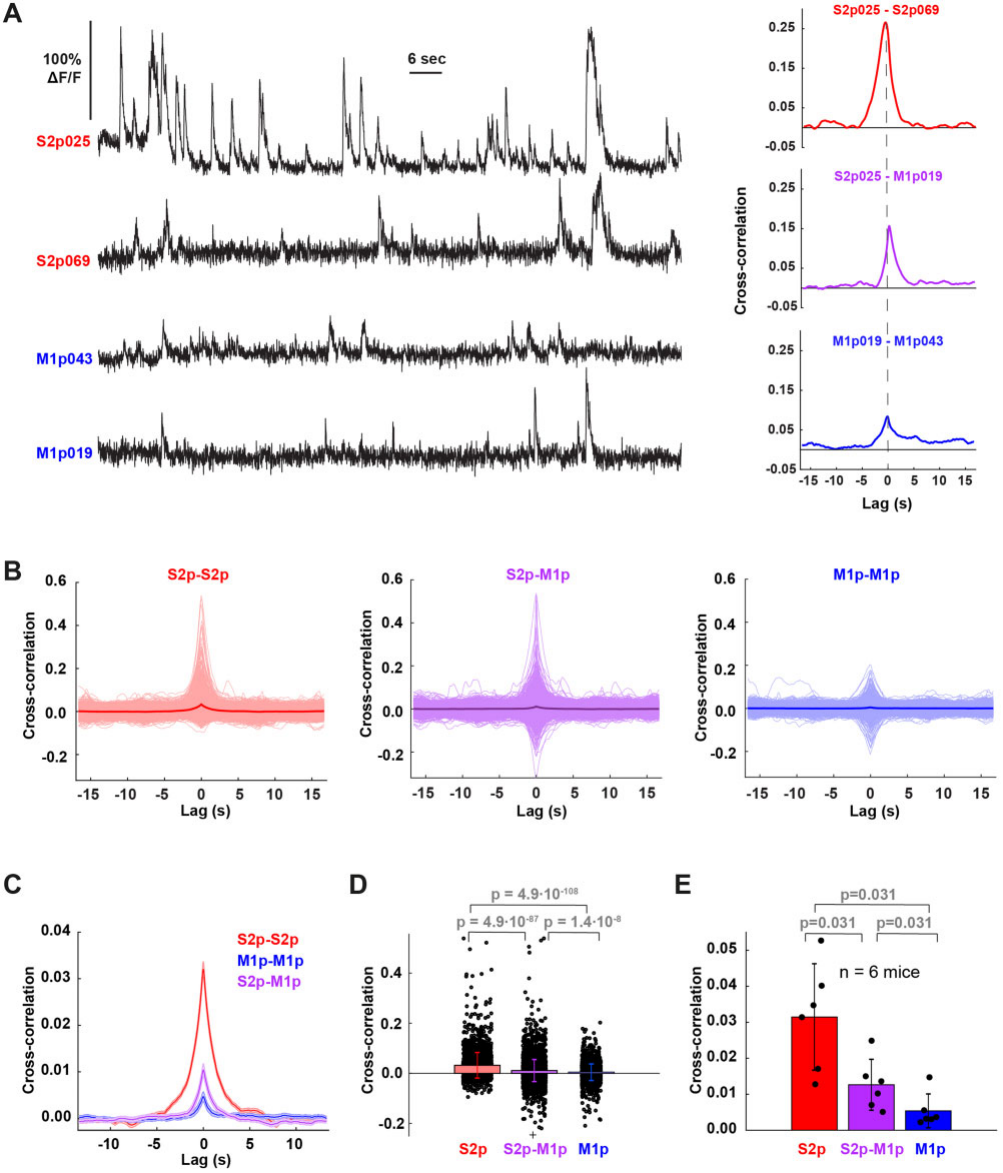
Cross-correlation Analysis in Correct Rejection Trials. (**A**) Left side, example of calcium signals of two S2p and two M1p neurons (mouse AV208). Right side, cross-correlation between example pairs of these neurons. (**B**) Cross-correlation between all pairs of S2p (*n* = 2521 pairs), M1p (*n* = 2143 pairs), and S2p-M1p neurons (*n* = 4719 pairs) across the six mice (thin lines individual pairs; thick lines grand averages). (**C**) Grand average cross-correlation of all pairs of S2p-S2p, S2p-M1p and M1p-M1p neurons (thick line: mean, shading: ± SEM). (**D**) Bar graph of mean cross-correlation at zero-lag for all pairs of S2p-S2p, S2p-M1p, and M1p-M1p neurons. Individual circles in black indicate the mean cross-correlation at zero-lag for individual pairs of neurons across all mice. Black crosses on top and bottom indicate off-scale outliers. Wilcoxon rank-sum test was used for statistical comparisons. Bar graphs with error bars represent the mean ± standard deviation. (**E**) Bar graph of mean cross-correlation at zero-lag for pairs of S2p-S2p, S2p-M1p, and M1p-M1p neurons across mice (*n* = 6). Individual filled circles in black indicate the mean cross-correlation at zero-lag for all pairs of neurons in a single mouse. Wilcoxon signed-rank test was used for statistical comparisons. Bar graphs with error bars represent the mean ± SD.

We next investigated the degree of correlated neuronal activity among simultaneously imaged neurons during correct rejection trials, in which no stimulus was delivered and the mouse did not lick. Spontaneous neuronal activity (Figure 8A) was found to be more strongly correlated among simultaneously imaged S2p neurons compared to among M1p neurons, with an intermediate correlation found between S2p versus M1p neurons (Figure 8B, C). Computed across all neuronal pairs, the zero time-lag correlation among S2p neurons (0.032 ± 0.051) was significantly (*P* = 4.9 x 10^−108^) larger than the correlation among M1p neurons (0.004 ± 0.033) (Figure 8D). Similar results were found when the correlations were calculated across the six mice (Figure 8E).

## Discussion

Through two-photon calcium imaging of retrogradely labeled layer 2/3 projection neurons in wS1, we found that S2p neurons had larger sensory-evoked responses, larger excitation preceding spontaneous licking, and more correlated spontaneous activity compared to the intermingled M1p neurons. Our results are consistent with the hypothesis that signaling from wS1 to wS2 might be important for whisker detection task performance.^13^^,^^21^^,^^22^

### Target-specific Activity of Layer 2/3 Projection Neurons

We previously measured membrane potential in S2p and M1p neurons during a closely related whisker detection task,^21^ finding enhanced depolarization in S2p compared to M1p neurons in whisker hit trials, and during spontaneous licking. Here, using two-photon calcium imaging of a much larger sample of projection neurons, we find results that are entirely consistent with the earlier electrophysiological study. The current study has three further advantages: (1) the S2p and M1p neurons are imaged simultaneously, and thus during identical mouse behavior; (2) the neurons were imaged across days revealing relatively stable representations; and (3) we introduced a second rewarded sensory stimulus, allowing to compare the response of S2p and M1p neurons to another sensory modality.

Our results are also in good agreement with a previous two-photon calcium imaging study during a whisker detection task, in which S2p neurons, compared to nearby unlabeled neurons, were found to more reliably encode the whisker stimulus and the lick probability.^13^ In our study, we found that S2p neurons were more responsive to both whisker and auditory stimulation compared to nearby M1p neurons, and that S2p neurons responded more strongly than nearby M1p neurons when the mouse licked spontaneously. Thus, S2p neurons, compared to M1p neurons, seem to more robustly encode the decision of the mouse to lick in whisker detection tasks. Distinct whisker sensory processing in S2p and M1p neurons has also been reported during texture discrimination tasks,^10^^–^^12^ with S2p neurons having more prominent decision-related signals compared to M1p neurons. Furthermore, *in vivo* whole-cell recordings from S2p and M1p neurons have noted important differences in excitability, spontaneous membrane potential fluctuations, and whisker touch processing also in naïve mice,^26^ so it is perhaps not surprising that these projection neurons also differ during task performance. Indeed, gene expression studies of S2p and M1p neurons reveal profound differences, suggesting that they might be different cell types.^38^

Similarly, studies of layer 2/3 projection neurons in the mouse visual system also found evidence for target-specific functional activity, with signals from V1 to downstream targets showing distinct spatial and temporal frequency preferences.^39^^,^^40^ Taken together, a growing body of work begins to suggest the overall importance of investigating functional activity of layer 2/3 neurons in the context of their long-range projection targets, similar to the prominent projection-specific differences found for mouse layer 5 neurons during task performance.^41^^,^^42^

### Neuronal Circuits for Goal-directed Sensorimotor Transformation

**Figure 9.**
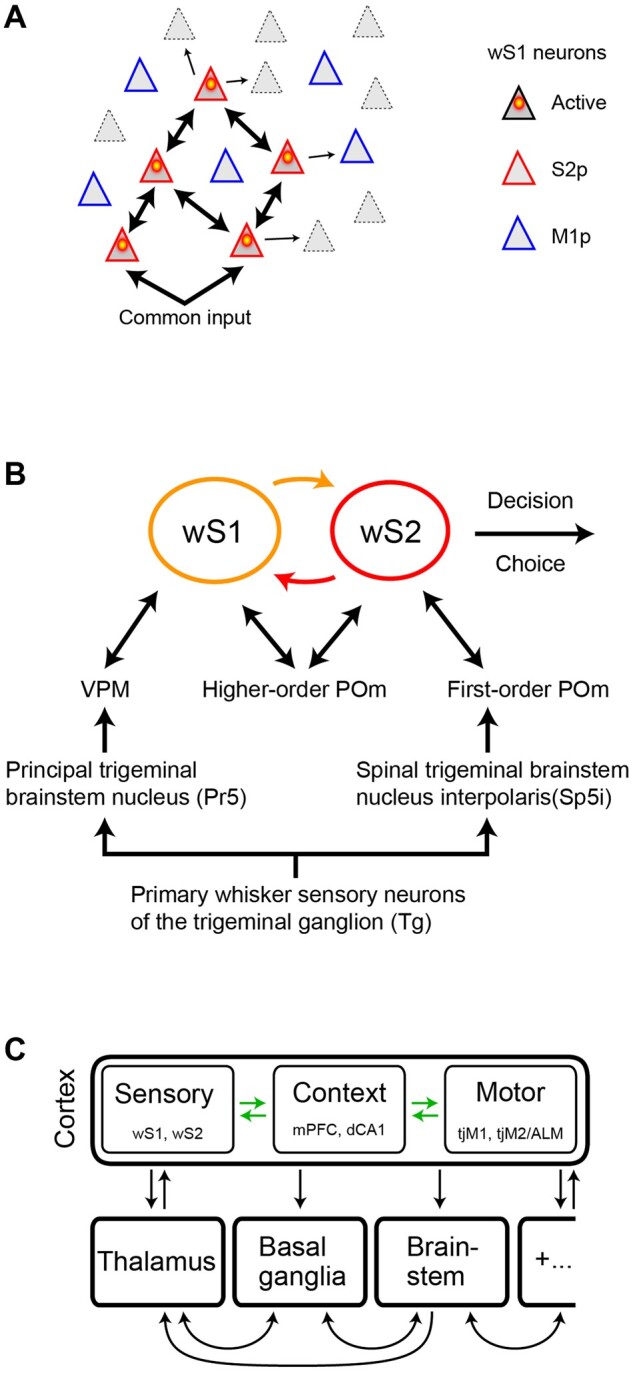
Some Possible Neural Circuit Mechanisms Contributing to the Execution of the Whisker Detection Task. (**A**) In the detection task, S2p neurons compared to M1p neurons respond more strongly to whisker stimulation and show enhanced correlated spontaneous activity. This could result from strong common synaptic input to S2p neurons and/or from strong reciprocal local excitatory synaptic connectivity between S2p neurons. (**B**) Whisker deflection evokes action potential firing in the primary sensory neurons of the trigeminal ganglion (Tg). The Tg neurons release glutamate on postsynaptic neurons in various trigeminal brainstem nuclei. The lemniscal pathway originates in the principal trigeminal nucleus (Pr5), which excites neurons in the ventral posterior medial (VPM) primary whisker somatosensory thalamic nucleus through glutamatergic synapses. The paralemniscal pathway originates from glutamatergic neurons in the spinal trigeminal interpolaris nucleus (Sp5i) of the brainstem which innervates an anterior first-order division of the posterior medial thalamus (POm). Glutamatergic projection neurons in VPM and POm in turn predominantly innervate wS1 and wS2 respectively. Reciprocal excitatory interactions through glutamatergic synapses between wS1 and wS2 may be a critical early step in whisker sensory perception and decision-making (13,44). Interactions of wS1 and wS2 with higher-order POm may also be important. (**C**) Neuronal activity in various brain areas likely contributes to converting the whisker sensory signal into the licking motor response. In addition to wS1 and wS2, further important nodes have been suggested to include additional cortical regions such as motor cortex (tjM1, tjM2/ALM), medial prefrontal cortex (mPFC) and the dorsal CA1 region of hippocampus (dCA1), as well as subcortical structures such as thalamus, basal ganglia and brainstem. Further research is likely to reveal additional participating brain areas (indicated by “+…”).

The current study supports the hypothesis that signaling from wS1 to wS2 might be important for execution of the whisker detection task. Neurons projecting to wS2 compared to those projecting to wM1 showed larger sensory-evoked responses and were also more strongly excited before spontaneous licking. The simultaneous imaging of many projection neurons allowed correlation analysis of spontaneous network dynamics, which showed that the activity of S2p neurons is more correlated than M1p neurons. Synchronous activity of S2p neurons could enhance their impact upon the postsynaptic targets in wS2, perhaps contributing to driving task execution. Highly correlated activity of S2p neurons (Figure 8) might result from strong reciprocal synaptic connectivity among these neurons and/or from strong common input to these neurons (Figure 9A). In the future, it will be of great interest to directly investigate possible differences in the synaptic connectivity of S2p and M1p neurons. Interestingly, a recent study suggested that strongly synaptically coupled neurons in wS1 contribute to amplification of sensory processing,^43^ which could also be relevant for the S2p neurons studied here.

Through axonal calcium imaging, Kwon et al.^13^ found evidence for an important contribution for reciprocal signaling between wS1 and wS2 during a whisker detection task. Furthermore, inactivation of either wS1 and wS2, but not wM1, reduces hit rates in the whisker detection task.^13^^,^^15^^,^^16^ Reciprocal interactions between wS1 and wS2 could help enhance and prolong activity evoked by the brief whisker deflection, which might contribute to the decision to lick through enhanced recruitment of downstream brain areas (Figure 9B). A further important future test of this hypothesis will be to specifically inactivate wS1 neurons projecting to wS2 or wM1 separately. Ideally, this would be achieved by inhibiting neurotransmitter release in the target region, to avoid interfering with the local circuit activity within wS1. Interestingly, thalamic input to wS2 relayed by the paralemniscal pathway, as well as higher order thalamic input to somatosensory cortices, was also shown to carry decision-related signals in a whisker detection task, raising the possibility of complex interactions between cortex and thalamus during decision-making^44^ (Figure 9B). Another important question for future investigations relates to which onward pathways from wS1 and wS2 might be responsible for the initiation of licking during learned whisker-detection task performance, with current data suggesting roles for additional cortical areas, including medial prefrontal cortex,^15^ dorsal hippocampal area CA1,^15^ licking-related tongue–jaw motor cortex (tjM1 and tjM2/ALM),^16^^,^^45^ as well as subcortical networks, such as the thalamus,^44^ basal ganglia,^19^ and brainstem nuclei (Figure 9C).

### Future Perspectives

Although two-photon imaging of neuronal activity with GCaMP6f has many advantages over whole-cell electrophysiological measurements, it currently has inferior temporal resolution and likely largely reports strongly responding neurons firing many action potentials. Improved calcium indicators, such as GCaMP7,^46^ GCaMP-X,^47^ and XCaMPs^48^ will likely help improve measurement accuracy. Furthermore, larger numbers of neurons could be imaged through volumetric 3D imaging,^8^ although at the expense of temporal resolution.

Further important future experiments include imaging of S2p and M1p neurons across whisker-detection task learning, to investigate if the properties of these neurons change.^11^^,^^21^ In addition, layer 2/3 neurons in wS1 project to multiple targets other than wS2 and wM1,^27^ and it will be important to differentiate the activity patterns of neurons taking into account the full diversity of axonal projections.

It will likely be necessary to measure, manipulate, and model projection-specific and cell-type-specific neuronal activity across multiple brain areas in order to fully understand the causal neuronal circuits underlying even relatively simple goal-direction sensorimotor transformations (Figure 9C). Ultimately, to uncover the mechanisms of learning, we need to understand how reward signals drive synaptic plasticity in specific neuronal circuits linking sensory input to the appropriate motor output, and optical methods, such as those deployed in this study, offer important opportunities for detailed circuit analysis. Exciting times ahead!
