# Corrigendum to: Projection-specific Activity of Layer 2/3 Neurons Imaged in Mouse Primary Somatosensory Barrel Cortex During a Whisker Detection Task

**DOI:** 10.1093/function/zqaa020

**Published:** 2020-10-06

Within the “Key Resources Table” the link for the “Software, algorithm” entry has been changed from https://zenodo.org/10.5281/zenodo.3911112 to https://doi.org/10.5281/zenodo.3911112. The same URL in the “Data and code availability” section has been amended as well.

